# Prevalence of self-reported fatigue in intensive care unit survivors 6 months–5 years after discharge

**DOI:** 10.1038/s41598-022-09623-w

**Published:** 2022-04-04

**Authors:** Jérôme Morel, Pascal Infantino, Laurent Gergelé, Thomas Lapole, Robin Souron, Guillaume Y. Millet

**Affiliations:** 1 Département Anesthésie Réanimation, Centre Hospitalier Universitaire de Saint-Etienne, Saint-Etienne, France; 2 Université de Lyon, UJM, Inter-university Laboratory of Human Movement Biology, EA 7424, 42023 Saint-Etienne, France; 3 Ramsay Générale de Santé, Hôpital Privé de la Loire, Saint-Etienne, France; 4 Nantes Université, Movement-Interactions-Performance, MIP, UR 4334, 44000 Nantes, France; 5 Institut Universitaire de France (IUF), LIBM, IRMIS, Campus Santé Innovations, 10 rue de la Marandière, 42270 Saint-Priest en Jarez, France

**Keywords:** Fatigue, Outcomes research, Quality of life

## Abstract

Prolonged stays in intensive care units (ICU) are responsible for long-lasting consequences, fatigue being one of the more debilitating. Yet, fatigue prevalence for patients that have experienced ICU stays remains poorly investigated. This study aimed to evaluate fatigue prevalence and the level of physical activity in ICU survivors from 6 months to 5 years after ICU discharge using the Functional Assessment of Chronic Illness Therapy Fatigue (FACIT-F) and Godin questionnaires, respectively. Data from 351 ICU survivors (out of 1583 contacted) showed that 199 (57%) and 152 (43%) were considered as fatigued and non-fatigued, respectively. The median FACIT-F scores for fatigued versus non-fatigued ICU survivors were 21 (14–27) and 45 (41–48), respectively (p < 0.001). Time from discharge had no significant effect on fatigue prevalence (p = 0.30) and fatigued ICU survivors are less active (p < 0.001). In multivariate analysis, the only risk factor of being fatigued that was identified was being female. We reported a high prevalence of fatigue among ICU survivors. Sex was the only independent risk factor of being fatigued, with females being more prone to this symptom. Further studies should consider experimental approaches that help us understand the objective causes of fatigue, and to build targeted fatigue management interventions.

## Introduction

Fatigue refers to an “overwhelming and sustained subjective sense of physical, emotional, and/or cognitive exhaustion that is not related to recent physical activity”^1^. Fatigue may persist over time so that Krupp et al.^2^ defined fatigue as “a feeling present for any amount of time on 50% of days for more than 6 weeks, limiting functional activities and/or quality of life”. Prolonged stays in the intensive care unit (ICU), along with the use of some medication, mechanical ventilation and prolonged periods of immobilization in bed, may induce consequent physiological alterations that could lead to persistent fatigue^3^. Fatigue prevalence in ICU survivors remains poorly investigated. Yet, its evaluation is critical because it is one of the main barriers to physical activity after ICU discharge^4^, that is believed to largely impact rehabilitation processes and in fine the health-related quality of life (HRQoL)^4–6^.

Fatigue is commonly reported from days to several months after hospital discharge in ICU survivors^6–11^. In the first year after ICU discharge, more than 50% of ICU survivors reported lowered energy levels and fatigue^7,9^. Fatigue is usually measured through specific self-report questionnaires or scales. Using the Multidimensional Fatigue Inventory-20 (MFI-20) questionnaire 3 and 6 months following discharge, fatigue was reported in 54% and 49.5% of ICU survivors, respectively^10^. When assessed with the Lee fatigue scale, fatigue was found in 15.3% and 13.8% of ICU survivors 3 and 12 months post-discharge, respectively^12^. Recently, the Functional Assessment Chronic Illness Therapy Fatigue (FACIT-F) scale has been used on 56 ICU survivors, 1 year after ICU discharge^13^, with a mean score of 39 ± 10 (out of a total score of 52, the lower the score the greater the fatigue). This corresponds to a moderate degree of fatigue when compared to the mean score (around 44) obtained in a large sample (n = 1010) of the general American population^14^. In cancer patients, the cut-off point value for fatigue diagnosis with the FACIT-F scale was reported to be 34^15^. While the analysis of the literature gives mixed results regarding the prevalence of fatigue in ICU survivors, differences in population, ICU conditions and experimental characteristics (e.g. time between ICU discharge and questionnaire survey) may explain the variability in fatigue prevalence reported in the aforementioned studies. Although speculative, another reason might be that most of the studies did not apply multidimensional and valid measures of fatigue. Moreover, studies used short and variable time frames with small sample sizes, preventing a clear interpretation of long-term fatigue.

As these limitations prevent a clear analysis of self-reported fatigue in ICU survivors, the first aim of this study was to evaluate fatigue in a large cohort of ICU survivors with a validated questionnaire and to identify independent risk factors of post-ICU fatigue. The second aim of this study was to use a questionnaire reporting physical activity to shed light on the potential relationship between fatigue and the level of physical activity after ICU discharge. Our main hypothesis was that a large part of ICU survivors would experience high levels of fatigue and that self-reported fatigue will decrease with time from discharge (i.e. the longer the time from ICU discharge the lower the level of fatigue). Our secondary hypothesis was that highly fatigued ICU survivors would present lower levels of physical activity.

## Methods

### Participants

Adult ICU patients discharged between January 2013 and May 2018 (which corresponds to 6–70 months (i.e. 5.8 years) after ICU discharge at the time of inclusion, i.e. October 2018) in two French medical and surgical ICUs were considered for enrollment. To meet the inclusion criteria, patients had to be between 18 and 90 years old, to have been mechanically ventilated for at least 3 days during their ICU stay (in order not to consider patients in ICU after an elective surgery who represent 10–20% of the patients admitted in both ICUs) and to have had a Simplified Acute Physiology Score II (SAPS II) score ≥ 15 at ICU admission (French definition for ICU patients). Foreign patients were not included (yet we did not exclude any patient for this reason). This study was approved by the local ethic committee “Comité Ethique Territorial Terre d’Ethique” (IRBN842018/CHUSTE) and written informed consent was obtained from all participants. This research was performed in accordance with relevant guidelines and in accordance with internationally accepted principles for medical research (World Medical Association of Helsinki 1964, revised in 2013).

### Measures

Questionnaires associated with a detailed explanation of the study were mailed to the ICU survivors. Participants were encouraged to circle the response that was most applicable for each question. Participants were asked not to skip any items. A stamped envelope was joined to return the fully filled-out questionnaires. We did not mail any reminder letter.

*The FACIT-F* scale is a 13-item questionnaire developed in cancer patients^16^. A growing body of evidence supports the validity of the FACIT-F to evaluate fatigue in ICU patients^3,17^. We used the French original version of the FACIT-F available on the Functional Assessment of Chronic Illness Therapy website^18^. This questionnaire assesses fatigue and its impact on daily activities and functioning. It comprises items such as the level of fatigue, the level of tiredness, the level of weakness and the impact of all these sensations on the ability to perform a usual level of activity or to keep eating habits for instance. Five choices of responses are allowed for each item, ranging from “not at all” (score = 0) to “very much” (score = 4). The final score ranges between 0 and 52, with lower scores representing higher level of fatigue. A cut-off point of 34 has been proposed to clinically diagnose fatigue using the FACIT-F questionnaire^15^. Because cut-off values associated with specific questionnaires are sometimes too restrictive to identify real effects, and to keep a margin of confidence, we arbitrary identified a “grey zone” around the proposed cut-off score of 34. This “grey zone” was ± 1 point around the score of 34, meaning that patients with a score of 33, 34 or 35 were not included in the statistical analysis. Patients with a score ≤ 32 were classified as fatigued and patients with a score ≥ 36 were classified as non-fatigued.

*The Godin Leisure-Time Exer*cise questionnaire scores is a reliable, simple, and effective measure of physical activity level. This questionnaire enables the assessment of self-reported leisure-time physical activity  and gives information on the number of times one engages in mild, moderate, or strenuous leisure-time physical activity for durations of at least 15 min within a typical 7-day period^19^. A score is calculated by multiplying activity frequency ratings by a Metabolic Equivalent of Task coefficient for each type of activity. Resulting values were then summed to give a leisure score index (LSI) expressed in arbitrary units where higher scores represent higher levels of physical activity. To our knowledge, this questionnaire has been validated in English but not in French. A non-validated French translation of the Godin Leisure-Time Exercise questionnaire has thus been used in the present experiment, as it has been done in other studies with French participants (e.g.^20^).

We received permission for the appointed institution to use the FACIT-F questionnaire. To the best of our knowledge, no permission is needed to use the Godin Leisure-Time Exercise questionnaire (e.g.^21,22^).

### Statistics

Age, sex, type of ICU admission, SAPS II, SAPS II-modified score (i.e. SAPS II without considering the age), Glasgow score, treatments received in ICU, duration of mechanical ventilation, uses of extracorporeal membrane oxygenation or renal replacement therapy, duration of ICU stay and time since ICU discharge were obtained from each ICU databases. We used the year of discharge (i.e. 2013, 2014, 2015, 2016, 2017 and 2018) to perform our statistical analysis as this variable fit best with our model (see below). We also considered the number of months from hospital discharge to give a more detailed description of the relationship between fatigue prevalence and time since discharge.

Statistical analyses were performed using XLSTAT version 2015.2.01 (Addinsoft, New York, NY) and R software version 4.1.0 (2021-05-18) (Vienna, Austria) according to published guidelines (R: A language and environment for statistical computing. R Foundation for Statistical Computing, Vienna, Austria. https://www.R-project.org/). Data were presented as medians [interquartile range 25–75%] or numbers (percentages). Data from fatigued and non-fatigued ICU groups were compared using the Mann–Whitney test for continuous variables and the Chi^2^ test for categorical variables. Statistical significance was assumed at p < 0.05. Two different multivariate models were built. The first one was a multiple linear model aimed at identifying independent variables associating with the FACIT-F score as a quantitative variable. Variables were selected in a forward stepwise manner, including only variables with p < 0.15 in the corresponding univariate model. The β coefficient, its 95% confidence interval and the p-value were displayed. A second multivariate model, using logistic regression was designed to predict the binary condition for fatigued versus non-fatigued ICU survivors. The variables were selected in a forward stepwise manner, to minimize AIC (Akaike Information Criterion). The odd ratios (ORs) were displayed with their 95% confidence intervals and p-values.

## Results

### Response rate

**Figure 1:**
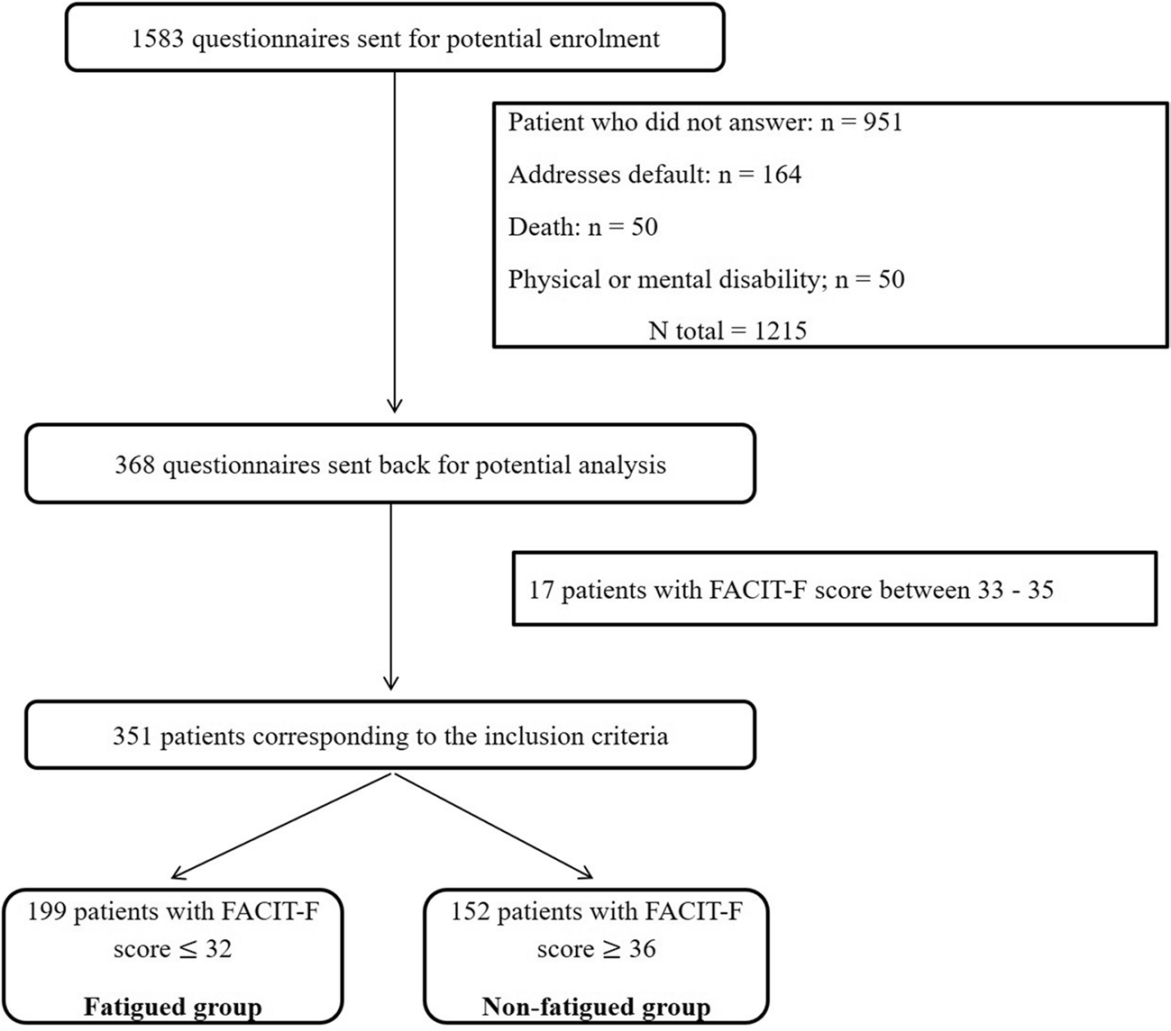
Flowchart of the study population.

Questionnaires were sent to 1583 patients, of which 368 replied (response rate: 23.1%). Among these 368 patients, 17 had a FACIT-F scores between 33 and 35 and were removed. Thus, data from 351 ICU survivors were included for statistical analysis (Fig. 1). No differences were found in the main ICU characteristics (ICU length of stay, age, SAPS II, sex ratio and type of admission or renal replacement therapy; p = 0.07–0.88) between the patients who returned the questionnaires (n = 351) and those who did not (n = 1215).

### Prevalence of fatigue

A total of 199 patients (57%) had a score ≤ 32 and were categorized as fatigued, while the remaining 152 (43%) had a score ≥ 36 and were categorized as non-fatigued. The median raw FACIT-F score of the entire studied population was 30.5 (20–43). Table 1 displays the main clinical characteristics and outcomes of both groups. The median FACIT-F score for fatigued and non-fatigued ICU survivors’ groups were 21 (14–27) and 45 (41–48), respectively (p < 0.001).

**Table 1:** Characteristics and outcomes of ICU survivors from the fatigued and non-fatigued groups.

	Fatigued ICU survivors (n = 199)	Non-fatigued ICU survivors (n = 152)	p-value
SAPS II score	40 (34–51)	44 (34–58)	0.09
SAPS II modified score	30 (22–39)	34 (24–46)	0.034
Male n (%)	110 (55)	112 (73)	< 0.001
Female n (%)	89 (45)	40 (27)	
Age	65 (52–73)	63 (52–69)	0.16
**Type of admission n (%)**			0.018
Surgical	143 (72)	91 (60)	
Medical	56 (28)	61 (40)	
**Admission diagnosis n (%)**			0.77
Respiratory	28 (14)	26 (17)	
Cardiovascular	35 (18)	23 (15)	
Gastrointestinal	4 (2)	2 (1)	
Neurological	53 (26)	36 (24)	
Trauma	10 (5)	4 (2.6)	
sepsis	38 (19)	34 (22)	
Others	31 (15)	27 (18)	
**Glasgow score at admission n (%)**			0.75
14–15	94 (47)	64 (42)	
9–13	13 (6)	11 (7)	
< 8	44 (22)	36 (24)	
Duration of mechanical ventilation (days)	8 (5–16)	7 (4–15)	0.37
ECMO n (%)	5 (2)	8 (5)	0.15
Renal replacement therapy n (%)	17 (8)	21 (14)	0.10
Length of stay in ICU (days)	14 (7–24)	12 (7–22)	0.15

Data are presented as median with interquartile range. Categorical data are reported as number with percentage in bracket.

*SAPS* Simplified Acute Physiology Score, *ECMO* extracorporeal membrane oxygenation, *ICU* intensive care unit.

**Figure 2:**
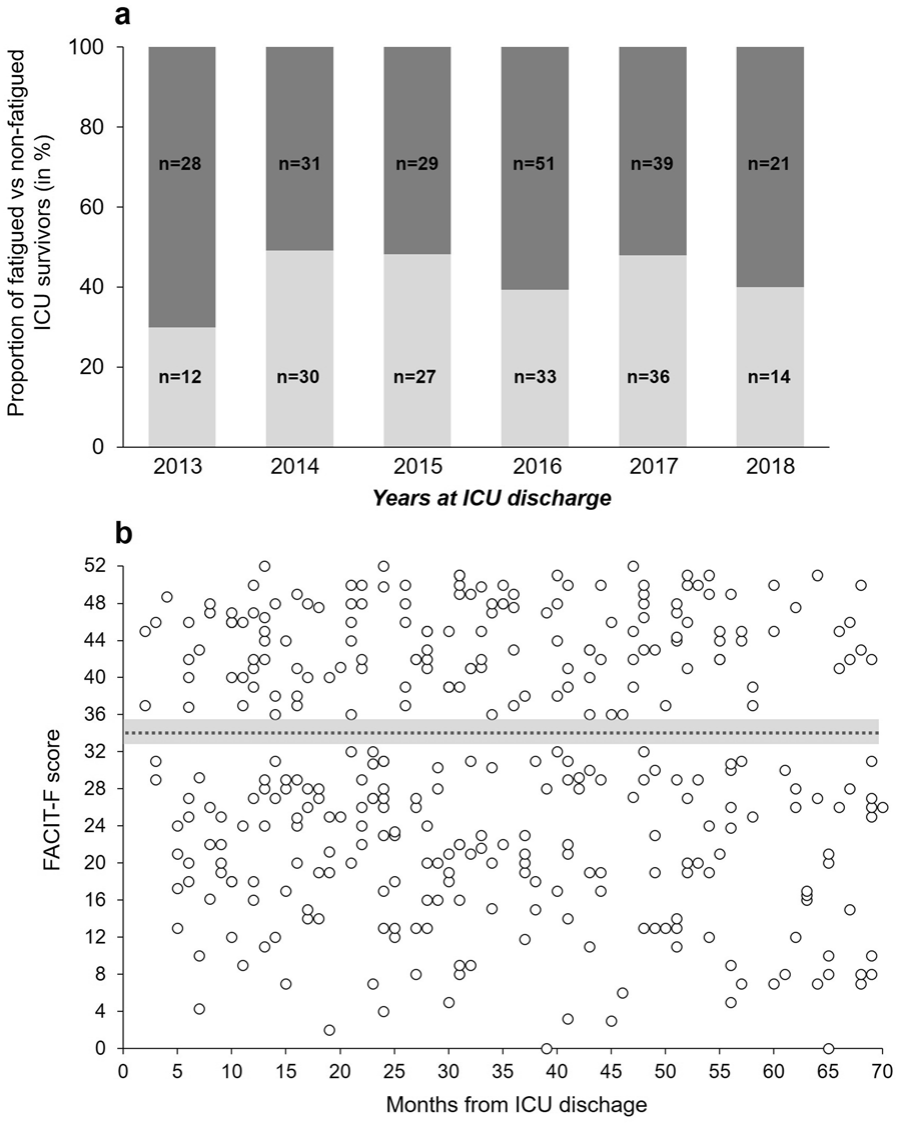
Fatigue prevalence in ICU survivors. (**a**) The proportion of fatigued (dark grey bar) versus non-fatigued (light grey bar) ICU survivors over the 5 years of the study period. (**b**) The FACIT-F scores of the 351 responders in connection with the number of months since ICU discharge. The dotted black line represents the cutoff point of fatigue (i.e. FACIT-F score = 34) proposed by Van Belle et al.^15^. Scores between 33 and 35 were ignored to clearly distinguish between fatigued and non-fatigued categories. These intermediate values are represented by the grey zone in (**b**).

The year of discharge had no significant effect on fatigue prevalence (Fig. 2A). Indeed, the proportion of fatigued patients was 70%, 51%, 52%, 61%, 52% and 60% in 2013, 2014, 2015, 2016, 2017 and 2018, respectively (p = 0.30). Figure 2B gives additional information on fatigue-related data distribution in relation to the number of months since ICU discharge.

Age of ICU survivors, SAPS II scores, duration of mechanical ventilation and length of stay were not statistically different between studied periods (p > 0.05; pooled data).

Fatigued ICU survivors were female in a larger proportion and were more frequently admitted for surgical reasons (Table 1). Duration of mechanical ventilation and ICU length of stay were not statistically different between fatigued versus non-fatigued groups. While SAPS II score was not significantly different between fatigued and non-fatigued ICU survivors (p = 0.09), a significant difference was found when the modified SAPS II score was considered (p = 0.03).

### Risk factors of fatigue

Length of stay, sex and age were independently associated with the FACIT-F score, meaning that FACIT-F score was lower (i.e. higher fatigue) in the oldest survivors, in females, and with an increased length of stay (Table 2). The SAPS II modified score was a significant predictor of fatigue in the univariate model but not in the multivariate one. In multivariate analysis, female sex was the only risk factor of being fatigued (Table 3).

**Table 2:** Multiple linear model associated to FACIT-F.

	β coefficient	CI 95%	p
No renal replacement therapy	3.5	[− 1.1; 8.0]	0.14
Length of ICU stay	− 0.11	[− 0.20; − 0.02]	0.02
SAPS II modified score	0.05	[− 0.03; 0.14]	0.23
Age	− 0.11	[− 2.33; − 0.20]	0.02
Sex (male)	4.65	[1.83; 3.24]	0.001
Type of admission (medical vs surgical)	2.29	[− 0.6; 5.18]	0.12

For each increase of 1 unit of the variable, there is an increase of the β coefficient.

*SAPS* Simplified Acute Physiology Score, *ICU* Intensive care unit.

**Table 3:** Multivariate logistic regression model for being in the fatigued group.

	OR	CI 95%	p
No renal replacement therapy	0.80	0.39–1.63	0.54
No ECLS	0.51	0.16–1.59	0.24
SAPS II modified score	0.99	0.98–1.0	0.38
Sex (male)	0.47	0.30–0.74	0.001
Type of admission (medical vs surgical)	0.65	0.41–1.02	0.06

Variables with an OR < 1 are protective.

*SAPS* Simplified Acute Physiology Score, *ECLS* emergency extracorporeal life support.

### Level of physical activity

Fatigued ICU survivors had a significantly lower level of physical activity when compared to non-fatigued ICU survivors, with a LSI score of 3.9 ± 8.8 versus 20.1 ± 20.0, respectively (p < 0.001). There was a significant correlation between FACIT-F and LSI scores (r = 0.48, p < 0.05).

## Discussion

This study reports that over half the ICU survivors (i.e. 57%) presented high levels of fatigue that was not dependent on the time from ICU discharge when assessed from 6 up to 70 months. While the length of stay in ICU, sex and age were independently associated with the FACIT-F score, being a female was the only factor independently associated with being fatigued. Finally, the present study shows that the fatigued ICU survivors are less active than their non-fatigued counterparts.

### Prevalence of fatigue in ICU survivors

Nearly 60% of ICU survivors reported being fatigued more than 5 years after discharge. This statement is highlighted by the fact that FACIT-F values (30.7 ± 13.4) in our studied population was far below the score of 44 ± 10 found in a large, general population sample^14^ and even below the score of 34 that has been reported to be of clinical relevance to determine fatigue^15^. Interestingly, the level of fatigue observed in this study is higher (i.e. lower FACIT-F score) than levels observed in other clinical settings, e.g. psoriatic arthritis (35.8 ± 12.4)^23^, inflammatory bowel disease (38.9 ± 11)^24^, yet it is not as low as in anemic cancer patients (23.9 ± 12.6)^14^.

As reported by two recent review articles^3,25^, there is a growing interest for the evaluation of fatigue prevalence in ICU survivors. However, the literature that brings direct evidence of fatigue prevalence in ICU survivors remains scarce, with many studies only investigating fatigue as a component of general HRQoL using questionnaires, e.g. 36-item Short Form Health Survey, SF-36^25,26^ (see below). FACIT-F was used in a small sample of ICU survivors (n = 56) 1 year after ICU discharge^13^. This study reported a median FACIT-F value of 41 (34–47) while it was 31 (23–42) in our study at the same time point. Some difference in inclusion criteria may explain these discrepancies. Of note, all the patients we included received mechanical ventilation for at least 72 h, while it was not the case in the study of Spadaro et al.^13^ with only 85% of the patients being mechanically ventilated. We made this choice because this specific condition leads to a state of advanced muscle deconditioning^27^, that may increase the risk of fatigue^10^. The link between fatigue and the duration of mechanical ventilation remains speculative at this stage, and future studies should investigate how this specific ICU intervention could impact the level of perceived fatigue. Fatigue starts early during the ICU stay, and 75% of the patients reported being fatigued when leaving ICU^9,28^. As shown in the present study, fatigue can persist for months or years after ICU discharge. In a large cohort of acute respiratory failure patients, the incidence of fatigue was around 70% 6 months after discharge with fatigue worsening in 28% of the cases at 12 months and fatigue was associated with physical, cognitive and mental health status^17^. Some authors reported that the results obtained for the “vitality domain” of the SF-36 were strongly correlated to the results obtained with the FACIT-F ^29^. Many studies showed a decrease of the vitality score of the SF-36 in ICU survivors^26^. These results give indirect evidence of a high level of perceived fatigue after ICU and corroborate the results obtained in the present work. However, such interpretation should be made with caution since i) no cut-off value has been established for the vitality domain and ii) the capacity of this score to accurately assess fatigue has been questioned^13^. Fatigue can also be assessed with a visual analogue scale. Using this tool, 45% of the subjects rated their fatigue as severe (≥ 6 over a scale of 10 points) at the end of their ICU stay^30^. The modified given symptom assessment tool reported that fatigue was highly prevalent in ICU survivors with 85%, 75% and 81% of patient reporting fatigue 2 weeks, 2 months and 4 months after discharge, respectively^8^. The sensitivity of visual analogue scales for evaluating fatigue is comparable to the sensitivity of various short and long questionnaires^31^, thus the aforementioned results could be easily confirmed.

In brief, the present work brings new and more solid evidence regarding the prevalence of fatigue in a large sample of ICU survivors, and over a long period (i.e. more than 5 years after discharge). This is problematic because a link between fatigue and long-term alterations in HRQoL has also been underlined directly or indirectly^10,32^, especially because high levels of fatigue limit exercise intentions and *in fine* reduces the general level of physical activity^4^.

### Factors associated with fatigue

Few studies investigated which ICU-risk factors (e.g. length of stay, duration of mechanical ventilation) or sociodemographic characteristics (e.g. age, sex) are associated with fatigue in ICU survivors. The presence of a coronary heart disease, the perceived fear of dying at ICU admission, major depression, and the number of comorbidities were found to be independently associated with post-ICU fatigue^10^. Our findings showed that women were more prone to fatigue than men. This sex effect on fatigue was also observed in other studies conducted in ICU survivors^10,17^ or in the general population^33^. As it stands, we do not have any clear explanation for this result, and further studies are warranted to shed light on this non-isolated result. We also confirm that the length of stay and patient age were independently associated with fatigue^17^. Unexpectedly, illness severity (i.e. SAPS-II score) at admission was not related with fatigue. We observed higher SAPS II modified scores (i.e. higher illness severity) in non-fatigued (34 [24–46]) than fatigued (30 [22–39]) ICU survivors, whereas we anticipated a higher score in fatigued patients. We found a similar trend in a recent study with COVID-19 patients (unpublished data). These results may appear counterintuitive. Indeed, it has been reported that higher SAPS II scores predict lower HRQoL scores within the first year after ICU discharge^34^. Because high levels of fatigue have a negative impact on HRQoL^35,36^, one would expect a relationship between fatigue and SAPS II score similar to the one reported between HRQoL and SAPS II score in this latter study^34^. However, other studies reported that SAPS II score did not correlate with 5-year HRQoL^37^. One should note that the abovementioned arguments were reported for the SAPS II (not modified) score. Whether this applies for the SAPS II modified score (i.e. SAPS II without considering the age) remains unknown, and any interpretation would remain purely speculative.

A sensitivity analysis ran with different fatigue thresholds (below and above 34) did not find any other significant risk factor of being in the fatigued group. Malnutrition is also frequent in ICU patients. Further research is needed to evaluate to what point malnutrition and other risk factors, such as the level of social integration for instance, can worsen fatigue, and whether the use of optimal care, or optimized nutrition may alleviate fatigue^38^.

### A need to consider targeted intervention to manage fatigue and improve recovery processes

Unrecognized and untreated fatigue may have negative effects on the patients’ HRQoL and rehabilitation process. We showed that ICU survivors affected by fatigue had lower levels of physical activity (i.e. lower LSI score), as it is the case for cancer patients with high level of fatigue^39^. Yet, it is difficult to determine if fatigue is the cause or the consequence of reduced physical activity.

Fatigue treatments encompass those that treat the initial disease justifying placement in ICU, as well as treatment of fatigue symptoms (e.g. weakness, sleep disorder, anemia, pain, depression). No study directly investigates the effectiveness of any treatment in preventing or reducing fatigue in ICU patients. ICU acquired weakness (ICUAW) may lead to long-term skeletal muscle dysfunction and reduction in physical capacities^32^. ICUAW is mainly explained by a loss of muscle mass^27^, and muscle wasting associated with prolonged ICU stay could play a major role in fatigue, as shown in other clinical conditions inducing cachexia^40^. Early rehabilitation programs that are effective in preventing muscle from wasting^3,41^ should start during the stay in the ICU and extend well after patient discharge to potentially alleviate fatigue. However, fatigue is a frequent barrier to exercise. Indeed, it has been suggested that fatigued patients may adjust their level of physical activity (i.e. minimize their daily energy expenditure) to maintain a self-perceived sensation of fatigue within a tolerable range^42^, which could reinforce neuromuscular deconditioning, thus limiting the compliance of ICU survivors to the rehabilitation programs^4,5^.

The conclusion of this process is an inevitable accentuation of fatigue months to years after ICU discharge. The results of the present study shed light on this major issue and encourage researchers and clinicians to improve ICU survivors’ HRQoL by reducing the level of fatigue. In particular, since the benefits of physical activity interventions to reduce the level of perceived fatigue have been proven (e.g.^43^), future studies should focus on designing tailored training programs to reduce fatigue after ICU discharge. It is essential that implementing anti-fatigue programs be considered early during the ICU stay, at the first stages of recovery during which fatigue can be experienced by patients^9,28,30^. This is especially the case for patients that develop the most severe neuromuscular alterations during their ICU stay. The use of specific assessment tools (e.g. ergometers that allow the assessment of evoked muscle forces using transcutaneous electrical/magnetic stimulation), as recently reviewed by our group^44^, could help to build targeted training programs that would reduce the prevalence of fatigue during the days and months that follow hospital discharge.

### Limitations of the study

One limitation of this study is the low response rate, i.e. ~ 23% of ICU survivors returned the questionnaires. No reminder mail was sent in the present study because we felt that doing so may have been unethical. However, it is important to note that this work is the first to evaluate the perceived sensation of fatigue in such a large cohort of ICU survivors (n = 351) and over such a long time period (>5 years after hospital discharge). Our results likely reflect what happened after ICU discharge in a representative sample of patients since we found no differences in the main ICU characteristics of the patients who returned the questionnaire and those who did not (see Supplemental Table 1). Fatigue is a complex symptom, and one should also note that there is no single measure of fatigue that can adequately capture its entire and complex nature. In addition, whether the fatigue experienced by survivors is entirely related to their ICU stay cannot be ascertained. However, we believe that by nature this is a limit common to all these types of studies, i.e. it is impossible to know the fatigue level of the patients before ICU admission. Further, the inclusion criteria (i.e. mechanical ventilation > 72 h and SAPS II > 15) limit the ability to evaluate the association between severity of illness and fatigue. Most of the patients were surgical, this selection bias may limit the extent to which our results can be generalized. As patient baseline status cannot be measured prior to ICU admission, it is possible that pre-existing impairments may have influenced our results. Others have shown that prolonged ICU stays lead to severe impairments in physical, psychological and cognitive outcomes, compared to status prior to ICU stay^45^, so we are confident that the fatigue-related results obtained in the present study are the consequences of the prolonged ICU stay (rather than any pre-existing impairments). Finally, data related to medication exposure (i.e. paralytics and sedatives) would have been of interest. Unfortunately, these data were not available.

## Conclusion

The incidence of fatigue was above 50% even more than 5 years after ICU discharge in a large sample of ICU survivors. This result should motivate clinicians to broadly assess various aspects (e.g. physical, cognitive, mental) of the health of ICU survivors due to their frequent co-occurrence with perceived fatigue. Furthermore, researchers and clinicians alike should consider longitudinal interventions and studies with a multifactorial approach to explain the etiology of fatigue, in particular the role of strength loss and muscle wasting. This could help to guide rehabilitation treatments and clarify the impact of fatigue on quality of life.

## Supplementary Information

Supplementary Table 1.

## Data Availability

All data generated or analyzed during this study are included in this published article (and its Supplementary Information files). The datasets generated during and/or analyzed during the current study are available from the corresponding author on reasonable request.
