# Supplementary material for: Prevalence of self-reported fatigue in intensive care unit survivors 6 months–5 years after discharge
Source: Sci Rep. 2022 Apr 4;12:5631. doi: 10.1038/s41598-022-09623-w (PMC8979153; doi:10.1038/s41598-022-09623-w)
Supplement: Supplementary file 1 — Supplementary Table 1. [file 41598_2022_9623_MOESM1_ESM.docx]

**Supplemental Table 1.** Characteristics of responders versus non-responders’ ICU patients.

| Parameters | Responders to questionnaire  N=368 | Non responders to questionnaire  N=1215 | **p-value** |
| --- | --- | --- | --- |
| ICU length of stay | 17.4 ±15.7 | 18.8 ±19.9 | **0.44** |
| Age | 61.0 ± 14.8 | 61.3 ± 16.0 | **0.88** |
| SAPS II | 44 ± 16 | 46 ±16 | **0.20** |
| Sex ratio (F/M) | 57.9% (135/233) | 55.2% (432/782) | **0.70** |
| Type of admission (S/M) | 246/122 | 740/475 | **0.07** |
| Renal replacement therapy (%) | 10.6 | 14.7 | **0.10** |

F: females; M: males; S: surgical; M: medical.
